# Factors associated with unintended pregnancy in Ethiopia; further analysis of the 2016 Ethiopian demographic health survey data

**DOI:** 10.1186/s12884-021-03924-0

**Published:** 2021-07-06

**Authors:** Yibeltal Alemu Bekele, Gedefaw Abeje Fekadu

**Affiliations:** Department of Reproductive Health and Population Studies, School of Public Health, Bahir Dar University, Bahir Dar, Ethiopia

**Keywords:** Unintended pregnancy, Ethiopia, Ethiopian demographic and health survey, Perceived distance

## Abstract

**Background:**

Unintended pregnancy an important public health problem in Ethiopia. It is associated with adverse physical, mental, social and economic outcomes. Identifying factors associated with unintended pregnancy may help to reduce unintended pregnancy and hence adverse outcomes. There are few studies about the prevalence and associated factors of unintended pregnancy in Ethiopia. But these studies were based on small sample size and fragmented. Therefore, this analysis was done to identify factors associated with unintended pregnancy in Ethiopia based on nationally representative data.

**Methods:**

The study used the 2016 Ethiopian demographic and health survey data. The data was downloaded from The DHS program with permission. A total of 1135 women were included in the final model. Data was weighted to consider disproportionate sampling and non-response. Multivariable logistic regression was used to identify factors associated with unintended pregnancy among women.

**Result:**

About 30% (95% CI: 25.33–34.39) pregnancies were unintended. Married women (Adjusted odds ratio (AOR); 0.34; 95% CI: (0.01–0.14), woman living in developing regions AOR; 0.14; 95% CI: (0.07–0.27) and women who reported distance was not a big problem to get medical care AOR; 0.59; 95% CI: (0.36–0.99) had lower odds of unintended pregnancy. On the other hand, multiparous AOR; 3.77; 95% CI: (1.71–8.33), grand multiparous AOR; 6.72; 95% CI: (2.74–16.49) women and women who ever used contraceptives AOR; 1.86 95% CI: (1.06–3.26) had higher odds of unintended pregnancy.

**Conclusion:**

Although high, the magnitude of unintended pregnancy in Ethiopia was lower compared to the global level. Marital status, region, perceived distance to seek medical care, parity and history of contraceptive use were found significant predictors of unintended pregnancy in Ethiopia.

## Background

Unintended pregnancy, defined as a pregnancy that is reported as either unwanted (pregnancy that occurred when no more children were desired) or mistimed (pregnancy that occurred earlier than the desired time). It is a global problem that affects the health of women, families and relatives. Unintended pregnancy occurs due to non-use or inconsistent uses of contraceptives or method failure [1, 2].

Globally, 44% of pregnancies were unintended in 2014. In developing countries, unintended pregnancy accounted for 65% of all pregnancies and 59% of these pregnancy ends with abortion. Currently unsafe abortion was among the main causes of maternal death globally. It contributes around 4.7–13.2% of maternal death every year worldwide [2–4]. About 1.9 million and 620,300 Ethiopian women had unintended pregnancies and abortions every year respectively. It represents an annual rate of 28 abortions per 1000 women ages 15 to 49 years [5–7].

Studies conducted in Kenya, Egypt, Nigeria and South Africa indicated that the prevalence of unintended pregnancies was 24, 30.7, 35.9 and 64.33% respectively [8–11]. Similar studies conducted in Ethiopia showed that the prevalence of unintended pregnancy ranged from 13.7 to 41.5% [12–19].

Studies conducted in African showed age, place of residence, marital status, ethnicity, types of employment, educational status, numbers of living children, monthly income and women’s autonomy, parity, gravidity, knowledge on contraceptives, accessibility of contraceptives were determinants of unintended pregnancy [8–11, 20]. Similarly, studies conducted in Ethiopia identified age, residence, religion, marital status, parity, visiting health professionals, history of abortion, age at first birth, family size, educational status, gravidity, distance from a health facility, history of stillbirth and knowledge of modern contraceptive methods as determinants of unintended pregnancy [12–19].

Despite improvements in contraceptive prevalence rate (CPR), the level of unintended pregnancy in Ethiopia was slightly declining from 42% in 2008 to 39% in 2014. Ethiopia planned to end all preventable causes of maternal mortality in 2030. Preventing unintended pregnancy is one of the key intervention areas to minimize maternal mortality [20–23].

In Ethiopia, various interventions were planned and performed to improve the contraceptive prevalence rate (CPR) and reduce the unmet need to minimize the impacts of unintended pregnancy. Identifying the level and associated factors of unintended pregnancy in Ethiopia was important to gauge the progress. Therefore, this analysis was done to identify factors associated with unintended pregnancy in Ethiopia based on nationally representative data.

## Methods

### Data

We used the 2016 EDHS data for this analysis. The 2016 EDHS is a community based cross-sectional data collected from January 18 to June 25, 2016. The survey was designed to provide key indicators at national and regional level. The survey used a two-stage stratified random sampling technique. First, each region was stratified into rural and urban areas. Then, enumeration areas (EA) were selected with probability proportional to enumeration area size. After this, household listing in the selected EAs was done. In the second stage, a fixed number of 28 households from each cluster were selected and included using systematic random sampling technique. In both surveys, women aged 15–49 who were either permanent residents of the selected households or visitors who stayed in the household the night before the survey were interviewed. An interviewer administered questionnaire was used to collect data. The details of the methodology; sampling technique, data collection and data quality assurance are available from EDHS reports [24]. A total of 15,683 reproductive age women were included in the survey. From these, 1135 women were pregnant at the time of the survey and included in the analysis.

### Measurement

The outcome variable for this analysis was unintended pregnancy, which had two categories (yes or no). The 2016 EDHS questionnaire asked all reproductive age women involved in the survey whether they were pregnant or not at the time of the survey. If the woman responded she was pregnant, then she was asked if the pregnancy was wanted at the time, wanted later or not wanted at all. When the woman reported the pregnancy was wanted at the time of the survey, the outcome variable was considered “no”, meaning the pregnancy was intended. On the other hand, if the woman reported the pregnancy was wanted later or was not wanted at all, the outcome variable was considered unintended and coded “yes.”

The independent variables included in this analysis were socio-demographic (age, educational status, marital status, household wealth index, residence, religion, working status of the woman and region), reproductive health (parity, history of abortion, history of previous contraceptive use and knowledge of contraceptives), access to health service (distance to health facility to seek medical care) and woman’s autonomy related variables (can refuse sex, refusing sex is justified if she suspects (STI).

**T**he wealth index is a composite measure of a household’s cumulative living standard. It measured according to household assets based on common goods, such as televisions, bicycles, materials used for house construction, domestic animal, land and other wealth-related characteristics. Factor scores of household assets were generated through a principal component analysis and then standardised and categorised into five quintiles poorest, poor, middle, rich, and richest family.

### Analysis

We used STATA software for this analysis. The data were weighted to adjust for over sampling or under-sampling and non-response. Descriptive statistics were calculated for all variables. Correlation between independent variables was checked before fitting the final regression model. Multivariable logistic regression analysis was done to identify factors associated with unintended pregnancy. When two independent variables were found to be correlated, one was dropped. In addition, complex survey analysis techniques were employed when computing odds ratios since DHS used a two-stage stratified sampling technique.

## Result

### Background characteristics of pregnant women

Five hundred fifty five (48.93%) respondents were in the age range of 25 to 34 years. Six hundred three (53.20%) respondents reported that they didn’t attend formal education. Two hundred fifty seven (22.67%) respondents were from households with the poorest wealth index. Majority of the respondents (96.59%) were married or living in union. Nine hundred seventy four (85.83%) respondents were rural residents. About three-fourth of these pregnant women were not working at the time of the survey (Table 1).

**Table 1:** Socio-demographic characteristic of pregnant women in Ethiopia, EDHS 2016, (*n* = 1135)

Variable	Frequency (%)
**Age**	
15 to 24	400.31 (35.26)
25 to 34	555.46 (48.93)
35 to 49	179.42 (15.81)
**Educational status**	
No education	603.93 (53.20)
Primary education	397.65 (35.03)
Secondary or higher	133.60 (11.77)
**Marital status**	
Single	38.73 (3.41)
Married/living union	1096.46 (96.59)
**Wealth index**	
Poorest	257.29 (22.67)
Poorer	264.88 (23.33)
Middle	213.03 (18.77)
Richer	198.13 (17.45)
Richest	201.84 (17.78)
**Residence**	
Urban	160.91 (14.17)
Rural	974.28 (85.83)
**Religion**	
Orthodox	364.94 (32.15)
Muslim	489.51 (43.12)
Other^a^	280.74 (24.73)
**Currently working**	
No	839.25 (73.93)
Yes	295.94 (26.07)
**Region**	
Major region	1019.86 (89.84)
City administrations	29.44 (2.59)
Developing region	85.88 (7.57)

^a^other include catholic, protestant, traditional and other none specified

### Reproductive health characteristics of pregnant women

Four hundred fifty six (40.23%) respondents were multiparous. One thousand twenty five (90.33%) respondents had no history of abortion. Six hundred seventeen (54.37%) respondents had never used contraceptives. Six hundred thirty five (56.01%) respondents reported distance was a big problem to get medical care. Six hundred fifty two (57.51%) respondents reported that they would not refuse sex when the husband request. One thousand one hundred six (97.49%) respondents were knowledgeable about contraceptives (Table 2).

**Table 2:** Reproductive health characteristics of pregnant women in Ethiopia, EDHS 2016, (*n* = 1135)

Variable	Frequency (%)
**Parity**	
Nulliparous	229.23 (20.19)
Primiparous	181.07 (15.95)
Multiparous	456.70 (40.23)
Grand multiparous	268.19 (23.62)
**History of abortion**	
No	1025.36 (90.33)
Yes	109.83 (9.67)
**Previous contraceptive use**	
No	617.17 (54.37)
Yes	518.02 (45.63)
**Knowledge about contraceptives**	
Know no method	28.51 (2.51)
Knows modern method	1106.68 (97.49)
**Distance to health facility for medical help**	
Big problem	635.78 (56.01)
Not a big problem	499.42 (43.99)
**Respondent can to refuse sex**	
No	652.89 (57.51)
Yes	482.30 (42.49)

### Magnitude of unintended pregnancy

The level of unintended pregnancy among Ethiopian women was 336 (29.66%) with (95% CI, 25.33–34.39). Unintended pregnancy was high in Oromia region and low in Harari region.

### Unintended pregnancy by characteristics of women

Among women who have unintended pregnancy, 169 (50.36%) respondents were in the age groups of 25 to 34 years. One hundred and ninety (56.69%) respondents who had an unintended pregnancy had not attained formal education. Among women who have unintended pregnancy, three hundred eight (91.63%) respondents were living in rural area (Table 3).

**Table 3:** Unintended pregnancies by women’s characteristics Ethiopia, EDHS 2016, (n = 1135)

Variables	Unintended pregnancy	
	Yes	No
Age		
15 to 24	100.35 (29.82)	299.96 (37.57)
25 to 34	169.14 (50.36)	386.32 (48.38)
35 to 49	67.21 (19.97)	112.21 (14.05)
Marital status		
Single	29.62 (8.81)	9.11 (1.14)
Married or living in union	307.09 (91.19)	789.38 (98.86)
Educational status		
No education	190.81 (56.69)	413.13 (51.74)
Primary	119.62 (35.54)	278.04 (34.82)
Secondary or higher	26.28 (7.81)	107.32 (13.44)
Resident		
Urban	28.18 (8.37)	132.73 (16.62)
Rural	308.52 (91.63)	665.76 (83.38)
Region		
Major region	326.62 (97.05)	693.24 (86.82)
City administrations	5.41 (1.61)	24.04 (3.01)
Developing region	4.67 (1.39)	81.21 (10.17)
Perceived distance to seek medical care		
Big problem	224.31 (66.65)	411.47 (51.53)
Not a big problem	112.39 (33.35)	387.02 (48.47)
Parity		
Nulliparous	36.41 (10.84)	192.82 (24.15)
Primiparous	47.91 (14.24)	133.16 (16.68)
Multiparous	143.90 (42.76)	312.79 (39.17)
Grand multiparous	108.48 (32.23)	159.71 (20)
History of Abortion		
No	301.61 (89.58)	723.75 (90.64)
Yes	35.09 (10.42)	74.73 (9.36)
Ever contraceptive used		
No	156.08 (46.38)	461.09 (57.75)
Yes	180.62 (53.67)	337.39 (42.25)

### Factor associated with unintended pregnancy in Ethiopia

On the multivariable analysis, marital status, region, perceived distance to seek medical care, parity and history of contraceptive use were found significantly associated with unintended pregnancy.

Women who were married had 66% lower odds of unintended pregnancy compared to women who were single, divorced and widowed AOR; 0.034; 95% CI: (0.01–0.14). Women who were living in developing regions were 84% less likely to have unintended pregnancy compared to women who were living in major regions AOR; 0.14, 95% CI: (0.07–0.27). Women who reported distance to health facility was not a big problem to get medical care were 41% AOR 0.59; 95% CI: (0.36–0.99) less likely to have unintended pregnancy compared to those who reported distance was a big problem to seek medical care (Table 4).

**Table 4:** Factors associated with unintended pregnancy in Ethiopia, EDHS 2016 (weighted),(n = 1135)

Variable	COR(95%CI)	AOR (95% CI)
**Marital status**		
Single	1	1
Married or living in union	0.12 (0.44–0.32)	0.034 (0.01–0.14)
**Educational status**		
No education	1	1
Primary	0.93 (0.62–1.41)	1.3 (0.74–2.06)
Secondary or higher	0.53 (0.26–1.06)	1.69 (0.62–4.67)
**Resident**		
Urban	1	1
Rural	2.18 (1.18–4.01)	1.59 (0.59–3.67)
**Region**		
Major region	1	1
City administrations	0.47 (0.24–0.95)	1.00 (0.37–2.67)
Developing region	0.12 (0.07–0.03)	0.14 (0.07–0.27)
**Perceived distance to seek medical care**		
Big problem	1	1
Not a big problem	0.53 (0.34–0.83)	0.59 (0.36–0.99)
**Religion**		
Orthodox	1	1
Muslim	0.20 (0.73–1.99)	1.23 (0.74–2.06)
Other^a^	1.11 (0.67–1.84)	1.05 (0.60–1.84)
**Parity**		
Nulliparous	1	1
Primiparous	1.91 (0.80–4.53)	2.37 (0.91–6.14)
Multiparous	2.44 (1.20–4.94)	3.77 (1.71–8.33)
Grand multiparous	3.59 (1.76–7.35)	6.72 (2.74–16.49)
**History of Abortion**		
No	1	1
Yes	1.12 (0.61–2.07)	1.03 (0.53–2.01)
**Ever contraceptive used**		
No	1	1
Yes	1.58 (0.96–2.60)	1.86 (1.06–3.26)

^a^other includes catholic, protestant, traditional and other none specified

The odds of having unintended pregnancy among multiparous and grand multiparous women (women with five or more births) was AOR; 3.77, 95% CI: (1.71–8.33) and AOR; 6.72, 95% CI: (2.74–16.49) respectively times higher compared to nulliparous women. Similarly, women who ever had used contraceptives had AOR; 1.86; 95% CI: (1.06–3.26) times higher odds of having unintended pregnancy compared to those who never used (Table 4).

## Discussion

The prevalence of unintended pregnancy in the study area was 29.66%, (95% CI: 25.33–34.39). The finding of this study was in line with studies conducted in Maichew Ethiopia [17] and Addis Zemen Ethiopia [18]. However, the finding was lower than studies conducted in Arsi Negele and Jimma Ethiopia [12, 14], Nigeria [10] and South Africa [11]. This might be due to the low contraceptive prevalence rate in Nigeria compared to Ethiopia [24–26]. The difference compared to the study in South Africa might be the difference in the proportion of married women in these studies; only 36% of sexually active women were married or living in a union in South Africa compared to 65% in Ethiopia [24, 27]. The presence of unmarried sexually active women may increase the risk of unintended pregnancy. The finding of this study was higher than studies conducted in Belesa Ethiopia, Addis Zemen Ethiopia [16, 18] and Kenya [8]. This might be due to differences in the study area. The difference compared to the study in Kenya might be the high contraceptive prevalence rate in Kenya compared to Ethiopia [28].

Married or in-union women had lower odds of having an unintended pregnancy. This finding was consistent with studies conducted in Ethiopia [15, 19], South Africa [11] and Kenya [8]. This may be due to pregnancy before marriage is not socially acceptable and may have an economic impact on the woman. Due to these reasons, the pregnancy is likely to be unintended [29]. In addition, women may fear facing the economic burden for rearing the baby alone may lead to unintended pregnancy [30].

Mothers who live in developing regions had lower odds of having an unintended pregnancy compared to those living in major regions. This finding was consistent with a study conducted in Ethiopia [31]. The reason for this might be due to the low demand for access to family planning in developing regions (Afar, Somali, Benshangul Gumuz and Gambela) [24]. Moreover, fertility preference among women in these regions was higher compared to those in other regions [32]. This implies that the government needs to work intensively on availability and accessibility of family planning services to reverse it impacts especially on developing region.

Mothers who reported distance was not a big problem to seek medical care had 41% lower odds of having unintended pregnancy compared to women who reported distance as a problem. This finding was consistent with studies conducted in Ethiopia [15, 33]. This might be related to access to family planning services. When women did not perceive distance as a problem, they may tend to use contraceptives [34]. In addition, it increases the indirect cost of the family planning service utilization like transportation cost and loss from other productive activities [35, 36].

Multiparous women had higher odds of having an unintended pregnancy compared to nulliparous women. This finding was consistent with studies conducted in Addis Zemen [18], Arsi Negele Woreda [12] and Debre Brhan town [15] and Kenya [8]. The reasons for this might be fertility preference among multipara women is lower than nulliparous. Therefore, the pregnancy among multiparous women is more likely to be unintended.

Women who ever used contraceptives had higher odds of having an unintended pregnancy compared to their counterparts. The finding of this study was in line with studies conducted in Ivory Coast [37] but contradict with study in Legabo Woreda, North East Ethiopia [38]. The reason for this may be women who ever used contraceptives were not using it just before the pregnancy occurred. In addition, women who ever used contraceptives may have experienced side effects and method failure [39]. This study identified key factors associated with unintended pregnancy which could be used to design interventions to reduce unintended pregnancy in Ethiopia. However, social desirability bias may have affected the results of this study. Many women in Ethiopia rationalize the pregnancy and report as intended although the pregnancy was mistimed or unintended.

## Conclusion

Although still high, the magnitude of unintended pregnancy in Ethiopia was lower compared to the global prevalence. Marital status, living in developing regions, perceived distance to seek medical care, parity and history of modern contraceptive use was found predictors of unintended pregnancy. Unintended pregnancy prevention efforts should be strengthened among unmarried and multiparous women. Further study is needed to evaluate the quality of family planning programs since women who have ever used had more odds of unintended pregnancy in this study.

## Data Availability

Upon request, the data is available on The DHS program website at https://www.dhsprogram.com/data/available-datasets.cfm.

## Abbreviations

AOR: Adjusted odds ratio
CPR: Contraceptive prevalence rate
DHS: Demographic Health Survey
EA: Enumeration area
EDHS: Ethiopian Demographic Health Survey
STI: Sexually transmitted infection
